# Simple versus cross-mattress sutures for nondisplaced flaps of the maxillary molar region: a randomized controlled trial

**DOI:** 10.1186/s12903-022-02551-1

**Published:** 2022-11-16

**Authors:** Hossein Babazade, Farshad Vossough, Nafise Maftouhi, Shokoofeh Konarizadeh

**Affiliations:** 1 Department of Gingival Surgery, Faculty of Dentistry, Hormozgan University of Medical Sciences, Bandar Abbas, Iran; 2 Student Research Committee, Faculty of Dentistry, Hormozgan University of Medical Sciences, Bandar Abbas, Iran

**Keywords:** Sutures, Cross-mattress, Nondisplaced flap surgery, Maxillary, Molar

## Abstract

**Background:**

The type of suture used in periodontal surgery can affect post-surgical complications. This study aimed to compare simple with cross-mattress sutures for nondisplaced flaps of the maxillary molar region.

**Methods:**

This randomized controlled trial included 32 candidates of nondisplaced flap surgery of the maxillary molar region referred to the private office of a periodontist in Bandar Abbas, Iran from January 21 to May 4, 2020. First, the patients’ age, sex, and plaque index were recorded. Then, they were randomized into two equal groups. In the first group, the interdental suturing was done using simple sutures with 4–0 vicryl threads, and in the second group, interdental suturing was performed using cross-mattress sutures with the same threads. The primary outcome was suture time, including the duration of the first suture and the total duration of all sutures. The secondary outcomes were bleeding on probing and the requirement of supplementary sutures immediately after the surgery, as well as the gingival index (at suture removal and one month after surgery).

**Results:**

The two groups were comparable regarding age, sex, and plaque index. The first suture duration was significantly longer in the simple group compared to the cross-mattress group (*P* < 0.001); however, the total suture time did not differ between groups. Moreover, a significantly higher number of patients in the simple group required supplementary sutures (50% vs. 6.3%, *P* = 0.006). There was no significant difference between groups regarding bleeding on probing and gingival index (at suture removal and one month after surgery).

**Conclusions:**

Cross-mattress sutures were superior to simple sutures in terms of supplementary suture requirement for nondisplaced flaps of the maxillary molar region, while the two suturing techniques were alike regarding total suture time, gingival index, and probing on bleeding.

**Trial registration:**

Iranian Registry of Clinical Trials (IRCT), IRCT20191224045882N1. Registered 08/02/2020. Registered while recruiting, https://www.irct.ir/trial/44754.

## Introduction

The surgical phase of periodontal treatment is designed to control or eliminate the periodontal disease, correct the anatomical defect that can predispose the patient to periodontal diseases, lead to cosmetic disfigurement, or prevent the placement of prostheses, and devise implants to replace the removed teeth [1].

Currently, flaps are extensively used in periodontal surgeries. Based on the location of the flap after surgery, the flaps are classified into two groups, namely displaced and nondisplaced. Displaced flaps are sutured in a location other than their primary position, while nondisplaced ones are sutured at their primary location [2]. An essential factor in flap surgery, which contributes to the treatment results, is the suture and its type [3]. The principal goal of dental sutures is to maintain the flaps in the required position for better wound healing, minimal dehiscence in response to the region’s tension, and rapid closure to decrease the risk of post-surgical infection [4]. Many local and general factors affect oral wound healing. Local factors include would size and localization, postoperative bleeding, local anesthesia, infection, ischemia/hypo-perfusion, mechanical trauma, presence of necrotic tissue, and many others, while general factors include old age, nutritional deficiency, immunosuppression, and underlying diseases, such as diabetes, uremia, anemia, and cancer [5].

Several types of sutures are administered by dental surgeons, including simple, sling, anchor, horizontal, and mattress [4]. Cross-mattress sutures are modified horizontal mattress sutures in which the sutures are crossed to provide stronger tissue bridges. In fact, after placing the knots, the two loops of this type of suture cross each other and, to some extent, overlap [6]. In this study, it was hypothesized that for nondisplaced flaps of the maxillary molar region, cross-mattress sutures would be comparable to simple sutures in terms of duration and that cross-mattress sutures would require fewer supplementary sutures and lead to lower bleeding on probing and better gingival index.

## Methods

### Trial design

This study was a nonblinded, randomized controlled trial with two parallel arms.

### Participants

The current study included patients referred for gum surgery to the private office of a periodontist in Bandar Abbas, Iran, from January 21 to May 4, 2020. The inclusion criteria were age < 75 years, periodontitis with pocket depth > 5 mm, and being scheduled for periodontal surgery in the maxillary molar region with nondisplaced flap. The exclusion criteria were systemic diseases causing wound healing disorders such as diabetes, pregnancy, lactation, smoking, drug abuse, unfavorable plaque index (> 20%), clotting disorders, and unexpected events during suturing such as needle breakage, thread rupture, or thread knot. Patients who did not attend the office for follow-up were also excluded from the study.

### Interventions

In the simple group, the interdental suturing was done using simple sutures with 4–0 vicryl threads (absorbable, round-bodied, 1/2 circle, 37 mm, Supa Medical Co., Tehran, Iran), and in the cross-mattress group, interdental suturing was performed using cross-mattress sutures with the same threads. The involved dental papilla was between the distal aspect of the fifth and the mesial aspect of the sixth teeth. There were no vertical incisions or releases. The number of involved teeth or the flap extension was from the mesial aspect of the first premolar to the distal aspect of the second premolar. After surgery, all patients were advised to avoid chewing in the areas of surgery, alcohol and tobacco, and hot foods and drinks for at least one hour after surgery, rinse the oral cavity with mouthwash twice daily for two weeks starting from 24 h after surgery, apply ice packs to the face (side of the surgery) for the first 24 to 48 h, take only cold foods and liquids during the first 24 h, and stay hydrated. Naproxen 500 mg tablets were prescribed every 12 h for pain and discomfort. Amoxicillin capsules (500 mg) were prescribed for infection every eight hours for five days.

### Outcomes

The primary outcome was suture time, including the duration of first suture and the total duration of all sutures. The secondary outcomes were bleeding on probing, requirement of supplementary sutures, and gingival index. All patients were followed up for one month after the surgery. The gingival index was assessed at the time of suture removal (10 days after the surgery) and one month after surgery. The gingival index scores each site from 0 to 3, with “0” indicating normal and “3” indicating severe inflammation characterized by spontaneous bleeding, edema, and erythema [7]. The requirement of supplementary sutures was determined by examining the site immediately after the surgery. Supplementary sutures were applied in case of flap mobility, nonconformity of the papillae or other parts of the flap with the underlying tissue, and inadequate control of bleeding. Bleeding on probing was only assessed at the one-month follow-up.

### Sample size

The sample size was calculated as at least 16 patients in each group based on data from a pilot study with a total suturing duration (primary outcome) of 102.06 ± 8.27 s in the simple group and 110.40 ± 6.05 s in the cross-mattress group, α = 0.05 and β = 0.1 (power = 90%). The “sampsi” command from the Stata software (version 14.2) was used for sample size calculation.

### Randomization and implementation

First, the patients’ age, sex, and plaque index [8] were recorded. Then, they were randomized into two groups using the sealed envelopes method [9]. The sealed envelopes were provided to the investigator to ensure allocation concealment. Upon entrance of each patient into the study, the envelopes were shuffled, and one was selected for that patient.

### Blinding

Due to the nature of interventions, neither the patients nor the assessor was blinded to the groupings.

### Statistical methods

The Statistical Package for the Social Sciences (SPSS) software (version 25.0, Armonk, NY: IBM Corp., USA) was used for statistical analysis. Descriptive statistics, including mean, standard deviation, frequency, and percentage, were used to describe the variables. Based on the Kolmogorov–Smirnov normality test results, the independent t-test was used to compare continuous variables between groups. The Chi-squared test was used for the comparison of categorical variables between groups. *P*-values < 0.05 were regarded as statistically significant.

## Results

**Fig. 1:**
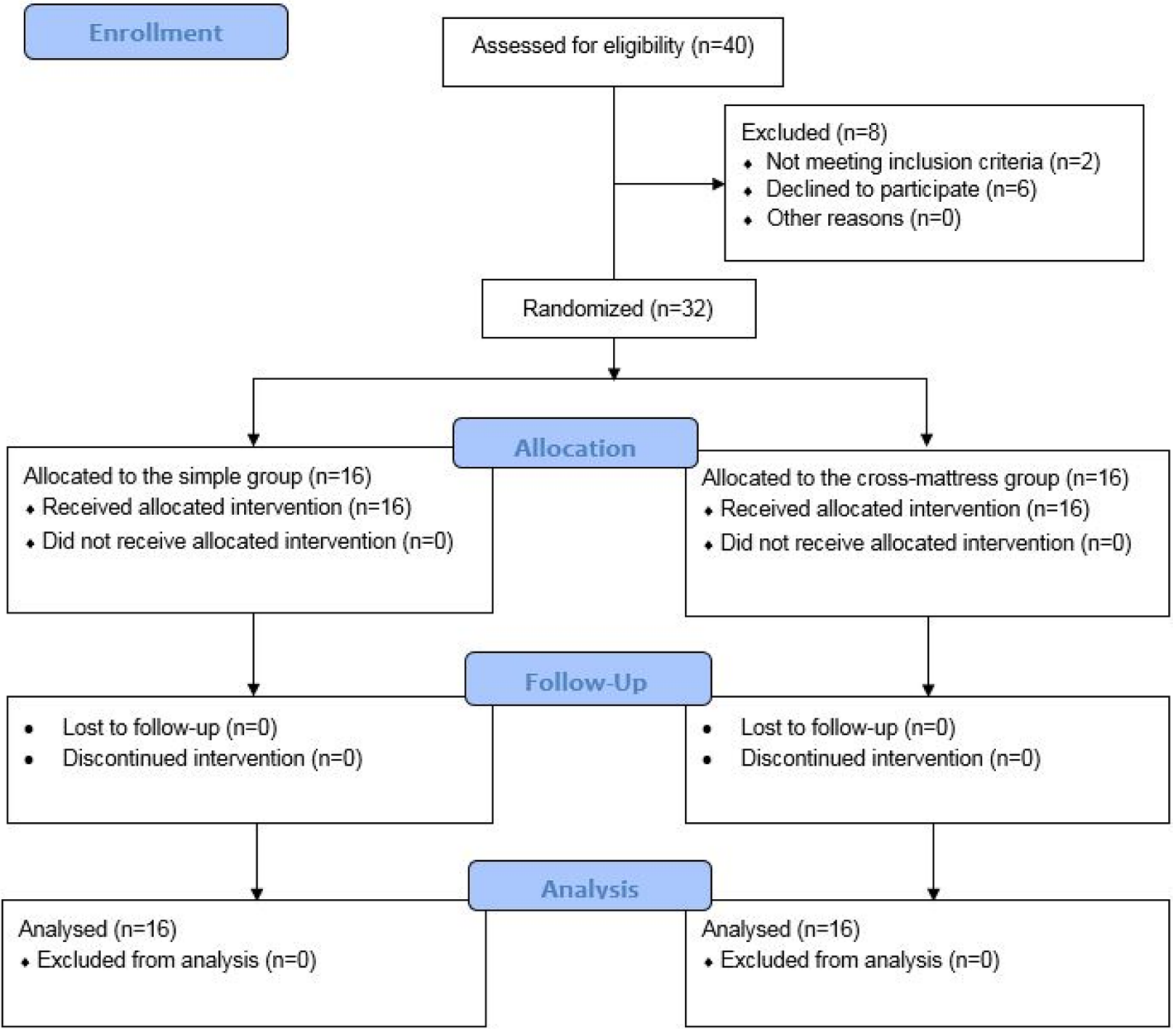
Details of patient enrollment, intervention allocation, and analysis

Initially, 40 patients were assessed for eligibility, of whom two did not meet the inclusion criteria, and six declined to participate in the study (Fig. 1). Of the remaining 32 patients, 21 (65.6%) were female, and 11 (34.4%) were male. Table 1 shows the general characteristics of the participants. There were no statistically significant differences between the two groups regarding age (*P* = 0.621), sex (*P* = 0.710), and plaque index (*P* = 0.658).

**Table 1:** Comparison of general characteristics between groups

Variables	Total (*n* = 32)	Simple (*n* = 16)	Cross-mattress (n = 16)	*P*-value*
Age (years), mean ± SD	42.78 ± 5.99	42.25 ± 7.41	43.31 ± 4.11	0.621
Sex, N (%)				
Male	21 (65.6)	10 (62.5)	11 (68.8)	0.710†
Female	11 (34.4)	6 (37.5)	5 (31.2)	
Plaque index (%), mean ± SD	14.87 ± 4.37	14.52 ± 4.88	15.21 ± 3.79	0.658

*Abbreviations*: *N* Number, *SD* Standard deviation

^* ^Analyzed by the independent t-test

^† ^Analyzed by the Chi-squared test

The first suture duration was significantly longer in the cross-mattress group compared to the simple group (*P* < 0.001), while total suture times were comparable between groups (*P* = 0.648). Supplementary sutures were required in a significantly higher proportion of patients in the simple group (*P* = 0.006). There was no statistically significant difference regarding bleeding on probing between groups (*P* = 0.476) (Table 2).

**Table 2:** Comparison of the first suture duration, total suture time, supplementary suture requirement, and bleeding on probing between groups

Variables	Simple (*n* = 16)	Cross-mattress (*n* = 16)	*P*-value*
Duration of the first suture (sec), mean ± SD	40.45 ± 3.72	50.17 ± 2.25	< 0.001
Total suture time (sec), mean ± SD	102.06 ± 41.31	107.44 ± 21.75	0.648
Supplementary suture requirement, N (%)	8 (50.0)	1 (6.3)	0.006†
Bleeding on probing, N (%)	8 (50.0)	6 (37.5)	0.476†

*Abbreviations*: *N* Number, *SD* Standard deviation, *sec* Seconds

^*^ Analyzed by the independent t-test

^†^ Analyzed by the Chi-squared test

Although gingival index scores of 2 and 1 were more frequently observed in the cross-mattress group at suture removal and one month after surgery, respectively, the difference between groups was not statistically significant (Table 3).

**Table 3:** Comparison of the gingival index at suture removal and after one month between groups

Gingival index N (%)	Simple (*n* = 16)	Cross-mattress (*n* = 16)	*P*-value*
At suture removal (10 days)			
3	4 (25.0)	2 (12.5)	0.365
2	12 (75.0)	14 (87.5)	
1	0 (0.0)	0 (0.0)	
One month after surgery			
3	0 (0.0)	0 (0.0)	0.476
2	8 (50.0)	6 (37.5)	
1	8 (50.0)	10 (62.5)	

*Abbreviations*: *N* Number

^*^ Analyzed by the Chi-squared test

## Discussion

In the current study, we compared simple with cross-mattress sutures for nondisplaced flaps of the maxillary molar region and found significantly longer first suture duration with cross-mattress but comparable total suture time between groups. Despite the longer duration of the first suture in the cross-mattress group, the similar total suture time in both groups shows that the overall suturing duration cannot be considered an advantage of simple over cross-mattress sutures. The simple suture is the most commonly applied technique in dentistry. The advantages of this technique include rapid and easy application [10, 11]. On the other hand, the cross-mattress suture enjoys the highest maximum load at failure due to the geometrical suture passage pattern and is considered biomechanically superior to the simple suture [6]. One disadvantage of cross-mattress sutures can be the longer suturing duration because of its more complex configuration than the simple suture. However, our study showed that the total suturing time was comparable between the simple and cross-mattress groups.

Another finding of our study was the significantly higher frequency of supplementary suture requirement in the simple group compared to the cross-mattress group, which may be due to the fact that suture opening can require less force in simple sutures. Similarly, Yamakado et al. have shown that a stronger force is needed for cross-mattress suture opening [6]. Furthermore, mattress suturing is basically applied in areas where closure cannot be performed without tension [12]. Also, the interdental space in the maxillary molar region is usually larger than to provide conformity of the soft tissue with the underlying bone using simple sutures [13]. Any extra manipulation of the oral cavity, such as supplementary sutures, can interfere with postoperative oral hygiene maintenance because they further disrupt the oral mucosa. Moreover, the disrupted oral mucosa may give rise to the vulnerability towards commensal microorganisms as well as pathogens [14], which in turn could affect the outcomes of patients regarding periodontal parameters.

We also found no statistically significant difference between the cross-mattress and simple groups in terms of the gingival index one month after surgery. On the contrary, Kumar et al. demonstrated a significant difference between simple and cross-mattress sutures regarding the gingival index [15]. Nonetheless, they performed their study on non-molar teeth, which can justify the discrepancy between their findings and ours. Optimal healing by placing and securing surgical flaps is the primary objective of suturing. Ideally, sutures should form secure knots, as well as be strong and handled easily [11].

In the present study, there was no significant difference between simple and cross-mattress sutures regarding bleeding on probing. Durability and stability of the flaps are major concerns in the postoperative period of flap surgery [16]. The oral cavity is mobile and moist, and the healing process occurs in a contaminated environment, which adds to the significance of periodontal surgery [17]. At the same time, the eating and speaking functions of the patients should continue, and some patients may smoke or have poor oral hygiene, which can adversely influence the healing process [18]. Altogether, these factors can contribute to the total outcome of surgery. However, suturing techniques can improve wound healing after surgery [19]. As shown by Kumar et al. and different from our results, modified vertical internal mattress sutures led to significantly lower bleeding on probing compared to simple loop interrupted sutures [15]. Of note, behavioral and environmental aspects, such as poor oral hygiene and cigarette smoking, can negatively influence wound healing and oral surgical outcomes [20]. However, we tried to eliminate these factors by advising all the patients to use mouthwash and avoid smoking in the postoperative period.

The present study was not without limitations. A major limitation was that we did not assess the gingival index at baseline; therefore, the effect of the baseline values on postoperative outcomes is unclear. Besides, pocket depth was not compared between groups pre- and post-operatively. On the other hand, the plaque index values of the side of the surgery were undetermined; a higher plaque index on the side of the surgery may have influenced the results. Another limitation was our inability to blind the patients and the assessor due to the nature of interventions in each group. Moreover, although none of the patients had infectious complications during the follow-up, we did not specifically assess post-surgical infections. Furthermore, we did not evaluate some potential factors affecting wound healing, such as smoking, food intake, nutritional status, and compliance with oral hygiene protocols.

## Conclusions

The results of the current study showed that cross-mattress sutures were superior to simple sutures in terms of supplementary suture requirement for nondisplaced flaps of the maxillary molar region. However, the cross-mattress technique was comparable to the simple suture technique regarding gingival index both at suture removal and one month after surgery. Also, the two suturing techniques were alike regarding total suture time and probing on bleeding.

## Data Availability

The datasets used and/or analyzed during the current study are available from the corresponding author on reasonable request.
